# Spatial Structure of the Mormon Cricket Gut Microbiome and its Predicted Contribution to Nutrition and Immune Function

**DOI:** 10.3389/fmicb.2017.00801

**Published:** 2017-05-12

**Authors:** Chad C. Smith, Robert B. Srygley, Frank Healy, Karthikeyan Swaminath, Ulrich G. Mueller

**Affiliations:** ^1^Department of Integrative Biology, University of Texas at Austin, AustinTX, USA; ^2^Northern Plains Agricultural Research Laboratory, Agricultural Research Service, United States Department of Agriculture, SidneyMT, USA; ^3^Department of Biology, Trinity University, San AntonioTX, USA

**Keywords:** symbiosis, alimentary tract, metagenomic prediction, phenol, carbohydrate metabolism, lactic acid bacteria, Enterobacteriaceae, katydid

## Abstract

The gut microbiome of insects plays an important role in their ecology and evolution, participating in nutrient acquisition, immunity, and behavior. Microbial community structure within the gut is heavily influenced by differences among gut regions in morphology and physiology, which determine the niches available for microbes to colonize. We present a high-resolution analysis of the structure of the gut microbiome in the Mormon cricket *Anabrus simplex*, an insect known for its periodic outbreaks in the western United States and nutrition-dependent mating system. The Mormon cricket microbiome was dominated by 11 taxa from the Lactobacillaceae, Enterobacteriaceae, and Streptococcaceae. While most of these were represented in all gut regions, there were marked differences in their relative abundance, with lactic-acid bacteria (Lactobacillaceae) more common in the foregut and midgut and enteric (Enterobacteriaceae) bacteria more common in the hindgut. Differences in community structure were driven by variation in the relative prevalence of three groups: a *Lactobacillus* in the foregut, *Pediococcus* lactic-acid bacteria in the midgut, and *Pantoea agglomerans*, an enteric bacterium, in the hindgut. These taxa have been shown to have beneficial effects on their hosts in insects and other animals by improving nutrition, increasing resistance to pathogens, and modulating social behavior. Using PICRUSt to predict gene content from our 16S rRNA sequences, we found enzymes that participate in carbohydrate metabolism and pathogen defense in other orthopterans. These were predominately represented in the hindgut and midgut, the most important sites for nutrition and pathogen defense. Phylogenetic analysis of 16S rRNA sequences from cultured isolates indicated low levels of divergence from sequences derived from plants and other insects, suggesting that these bacteria are likely to be exchanged between Mormon crickets and the environment. Our study shows strong spatial variation in microbiome community structure, which influences predicted gene content and thus the potential of the microbiome to influence host function.

## Introduction

Insects are the most speciose and abundant taxa in the animal kingdom, playing a key ecological role in many of the world’s ecosystems. Symbioses between insects and their microbial associates has undoubtedly contributed to their success, providing the capability to degrade recalcitrant food, to supplement nutrient-deficient diets, to protect them from their natural enemies, and to modulate the expression of social behavior (Engel and Moran, 2013; Douglas, 2015). Among the niches available to occupy the host, the gut often houses the largest and most diverse microbiome (Engel and Moran, 2013; Douglas, 2015). Gut morphology and physiology vary markedly along the alimentary tract, resulting in an environmental gradient that influences, and is influenced by, the microbial communities that populate it (Dillon and Dillon, 2004; Engel and Moran, 2013).

The insect gut consists of three regions that are analogous to that in mammals, the foregut, the midgut, and the hindgut, each of which contributes to a different aspect of gut function (Douglas, 2013). The foregut serves as the entry point for food, where it is stored in the crop before passing through the proventriculus, a valve that can also be modified to mechanically filter food (Woodring and Lorenz, 2007; Douglas, 2013) and even microbes (Lanan et al., 2016). Digestion and absorption of nutrients begins at the midgut, which, in some species, contains specialized crypts that house microbes that aid in insect nutrition (Kikuchi et al., 2005; Bistolas et al., 2014). Host immune factors have been shown to play an important role in regulation of commensal microbes in the midgut (Ryu et al., 2010; Buchon et al., 2013), some of which protect the host from pathogens (Forsgren et al., 2010). Following the midgut is the hindgut, which is comprised of the ileum, colon, and rectum. Malpighian tubules permeate the anterior hindgut, excreting nitrogenous waste and other solutes from the hemocoel that can provide nutrients for dense populations of microbes (Bignell, 1984). In some species, dense bristle-like structures in the ileum (Woodring and Lorenz, 2007) and rectal papillae (Hunt and Charnley, 1981) provide attachment sites for bacteria, some of which fix nitrogen (Tai et al., 2016), degrade recalcitrant plant polymers (Kaufman and Klug, 1991; Engel and Moran, 2013), and prevent infection (Dillon and Charnley, 2002).

The Mormon cricket *Anabrus simplex* (Orthoptera: Tettigoniidae) is an economically important shield-backed katydid distributed throughout the western United States. Mormon crickets can form dense aggregations of millions of individuals spread over 10 km long and several kilometers wide, feeding on forbs, agricultural crops, fungi, and invertebrates (including conspecifics) as they march in migratory bands across the landscape (MacVean, 1987; Simpson et al., 2006; Srygley, 2016). Mormon crickets are also emerging as a model for the study of how social interactions and diet influence immunity (Srygley et al., 2009; Srygley and Lorch, 2011) and the microbiome (Smith et al., 2016). Differences in population density are linked to reproductive behavior, as in high density populations, protein-limited females compete for access to males to gain access to a proteinaceous “nuptial gift” males produce for females during copulation (Gwynne, 1984). While consumption of male nuptial gifts by females does not influence the composition of the microbiome, sexually inactive females experience a dramatic decline in *Pediococcus* lactic-acid gut bacteria compared to sexually active females (Smith et al., 2016). Lactic-acid bacteria are common associates of the alimentary tract and regarded for their beneficial effects on immune function and nutrition in animals, including insects (Forsgren et al., 2010; Storelli et al., 2011; Erkosar et al., 2015).

We characterize the structure of the gut microbiome of Mormon crickets and infer their evolutionary relationships using a combination of culture-dependent and culture-independent approaches. Our aims are to determine whether gut microbial communities vary along the alimentary tract in the Mormon cricket and to infer their potential to influence host function based on their known taxonomic associations with other insects and predicted gene content from 16S rRNA sequences. We also establish methods for isolating Mormon cricket gut microbiota in culture to permit future experimental manipulations of the gut microbiome and build genomic resources to infer their evolution and function.

## Materials and Methods

### Animal Collection and Tissue Processing

Mormon crickets for 16S rRNA sequencing were obtained from field (*n* = 5) and laboratory-raised (*n* = 8) collections. Wild females were caught in EK Mountain (43°47′58″N, 106°50′31″W, 1752 m) near Kaycee, Wyoming in the summer of 2014, immediately preserved in 100% ethanol, and stored at -80°C until dissection. Laboratory-raised Mormon crickets were derived from eggs collected from individuals caught on Paint Rock Road (44°27′52″N, 107°27′37″W, 2654 m) in the Bighorn Mountains, WY and fed a mixture of wheat bran, wheat germ, sunflower, mixed bird seeds, tropical fish flakes, fresh Romaine lettuce (added daily), and water *ad libitum*.

**FIGURE 1:**
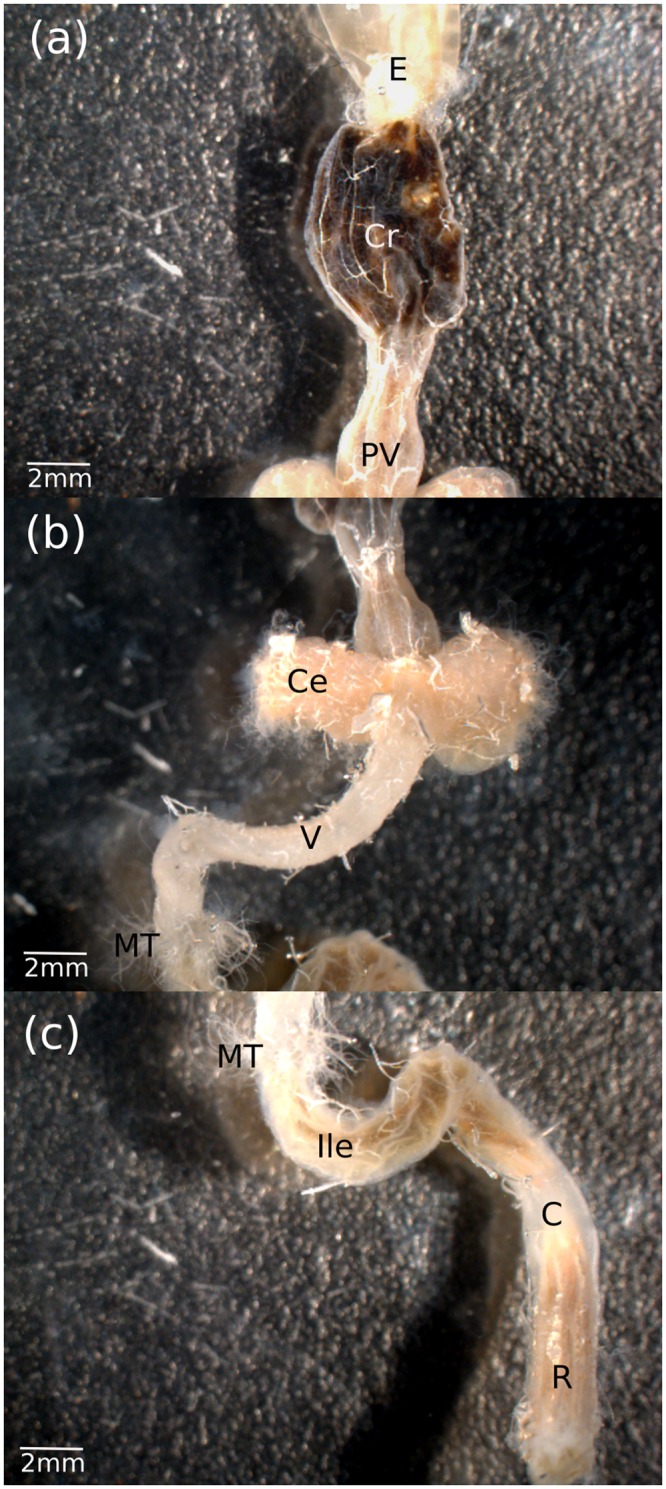
**External morphology of the (a)** foregut, **(b)** midgut, and **(c)** hindgut in the Mormon cricket. E, esophagus; Cr, crop; PV, proventriculus; Ce, cecum; V, ventriculus; MT, Malpighian tubules; Ile, ileum; C, colon; R, rectum. Malpighian tubules have been trimmed to illustrate their entry point into the hindgut.

Mormon crickets were dissected using flame-sterilized tools after rinsing in 1% bleach for 3 min followed by two rinses in autoclaved distilled water to remove bacteria on the exoskeleton. DNA from a 0.5 cm piece from the middle of the foregut (crop), a 0.5 cm piece from the middle of the midgut (ventriculus), and the entire ileum and rectum (**Figure 1**) of laboratory-raised crickets was extracted with a MoBio Powersoil© kit as in Smith et al. (2016). Foregut (crop), midgut (ventriculus), and hindgut tissue (ileum and rectum combined) was extracted from field-collected animals using a bead-beating/phenol–chloroform extraction protocol (see Supplementary Material). DNA extraction methods can influence the representation of taxa in 16S rRNA metagenomic studies (Yuan et al., 2012), however, our aim here is not to make inferences about differences between field and laboratory-raised animals but differences among tissue types. We include the source of the animal (field or laboratory) as a covariate in our statistical analyses to account for variation due to source/DNA extraction method (see Statistics).

### Sequencing and Bioinformatics

The variable V4 region of the 16S rRNA gene was amplified with universal primers (Hyb515F: 5′-GTGYCAGCMGCCGCGGTA-3′, Hyb806R: 5′-GGACTACHVGGGTWTCTAAT-3′) and sequenced on the Illumina Miseq V3 platform. DADA2 1.1.5 (Callahan et al., 2016) was used to process the raw sequencing data and taxonomy was assigned with the Greengenes 13.8 database at 97% identity (see Supplementary Material). Sequence variants that comprised an average of less than 1% of the reads recovered within a given Mormon cricket were removed prior to analysis using phyloseq 1.16.2 (McMurdie and Holmes, 2013).

### Metagenomic Predictions

We used PICRUSt (Phylogenetic Investigation of Communities by Reconstruction of Unobserved States) v1.1.0 (Langille et al., 2013) to estimate the functional gene content of our samples. PICRUSt generates metagenomic predictions from 16S rRNA data using annotations of sequenced genomes in the IMG database. Nearest sequenced taxon index (NSTI) values were small (mean ± SD: 0.03 ± 0.01, range: 0.006–0.040), indicating that the taxa in our samples were closely related to the genomes in the IMG database (see Supplementary Material). Greengenes IDs used by PICRUSt to construct the phylogenetic tree were assigned to sequence variants using Qiime 1.9 (Caporaso et al., 2010), and the Kyoto Encyclopedia of Genes and Genomes (KEGG) database was used for functional classification.

### Bacterial Abundance

We estimated the absolute abundance of bacteria in gut tissue from field-caught (*n* = 8) and laboratory-raised (*n* = 8) Mormon crickets using qPCR to compliment the information on relative abundance from Illumina sequencing (Props et al., 2017) following Powell et al. (2014). Universal 16S rRNA gene primers 27F (5′-AGAGTTTGATCCTGGCTCAG-3′) and 355R (5′-CTGCTGCCTCCCGTAGGAGT-3′) were used to amplify all 16S rRNA genes in each sample and copy number quantified using standard curves from the cloned target sequence (Promega, Madison, WI, USA; Powell et al., 2014) on an Applied Biosystems ViiA7 (Life Technologies). Triplicate 20 μl reactions were used with 10 μl of 2× Power SYBR master mix (Applied Biosystems), 0.4 μl of each 10 mM primer and 5 ng of template DNA. Template DNA concentration was normalized prior to qPCR using the Quant-iT Picogreen dsDNA Assay (Life Technologies) and an Infinite Pro M200 Pro microplate reader (TECAN). PCR amplification was performed at 95°C for 10 min followed by 40 cycles of 95°C for 15 s and 1 min at 60°C. Samples with melting curves that did not match that of the cloned target sequence were removed prior to analysis. Due to the large proportion of laboratory-raised samples that exhibited non-specific amplification (13/32 = 40.6% samples with non-specific amplification), we only present results from the field-caught individuals (2/32 = 6.3% samples with non-specific amplification).

### Culturing and Phylogenetic Analysis

Five lab-reared female Mormon crickets were surface sterilized in 1% bleach for 3 min, rinsed twice in sterile water and dissected using flame-sterilized tools. Gut tissue was homogenized for 10 s with a Biospec Mini-Beadbeater 96 using two autoclaved 3.2 mm stainless steel beads per tube in sterile PBS. Homogenates were plated onto trypsin soy agar, brain heart infusion agar, nutrient agar, or Man–Rogosa–Sharpe agar (BD), cultured in anaerobic or Campy (low O_2_) Gaspak pouches (Becton, Dickinson and Company, Franklin Lakes, NJ, USA) at 37°C for 24–48 h, and individual colonies passaged three times to obtain pure isolates. DNA was then extracted by boiling cells for 15 min in lysis buffer (100 mM NaCl and 0.5% sarcosyl), adding an equal volume of 20% chelex, and boiling for 15 additional minutes. The 16S rRNA gene was amplified for Sanger sequencing using 27F (5′-AGAGTTTGATCCTGGCTCAG-3′) and 1492R (5′-GGTTACCTTGTTACGACTT-3′) primers using Apex PCR master mix (Genesee Scientific, San Diego, CA) with 35 cycles (95°C for 20 s, 52°C for 1 min 30 s and 72°C for 40 s). PCR products were cleaned up with Sera-mag beads (GE Healthcare Life Sciences, Pittsburgh, PA) or ethanol precipitation and sequenced at the University of Texas at Austin on an Applied Biosystems 3730XL DNA analyzer.

We compiled 16S rRNA sequences from NCBI reported as sourced from insect guts (minimum size of 1.2 kb) and used BLAST to find the closest matches to our Mormon cricket isolates. *Pediococcus* and *Lactobacillus* sequences were aligned with pyNAST as implemented in Qiime 1.9 (Caporaso et al., 2010) using a curated alignment for *Lactobacillus* (McFrederick et al., 2013) as the reference template. The alignment was then manually edited with Mesquite (Maddison and Maddison, 2016) and filtered to remove characters with less than 80% coverage across sequences using Qiime 1.9 (Caporaso et al., 2010). Sequences from the Enterobacteriaceae were aligned with online implementation of the SILVA release 113 (Pruesse et al., 2012; Quast et al., 2013), manually checked in Mesquite (Maddison and Maddison, 2016), and filtered as above, with the additional removal of the top 10% most entropic (hypervariable) base positions. The phylogenies were constructed using maximum likelihood with a GTR + Gamma model for nucleotide evolution in RaxML 8.2.4 (Stamatakis, 2014), with 1000 bootstraps to assess branch support. Archaeopteryx 0.9916 (Han and Zmasek, 2009) was used to visualize the tree and produce the figures.

### Phenotypic Assays

Fresh overnight cultures of all isolates were used for microscopic analysis. Lactobacillaceae isolates were cultured in Man–Rogosa–Sharpe medium and Enterobacteriaceae were cultured in nutrient broth or LB medium. Biochemical tests were done following Bridson (1998). Motility was determined using SIM medium and microscopic examination of culture wet mounts. Man–Rogosa–Sharpe or nutrient broth containing 1 g/l potassium nitrate was used for nitrate reduction tests. Fermentation tests were done anaerobically in Man–Rogosa–Sharpe and nutrient broth media with the addition of indicated sugars to 1% w/v final concentration.

### Statistics

Analyses were performed in R 3.3.1 (R Core Development Team, 2013). Sequence tables were rarefied at 1300 reads using phyloseq (McMurdie and Holmes, 2013), resulting in the exclusion of hindgut samples from two field-caught females that had a low number of reads. Alpha diversity was compared among tissue types and between animal sources (field vs. lab) with a linear mixed model (Bates et al., 2013), entering the individual ID as a random effect to account for within-subject correlations in diversity. *Post hoc* comparisons among gut regions were performed using a Tukey test (Hothorn et al., 2008).

Beta diversity among gut tissue types and between animal sources (field vs. lab) was assessed with a distance-based redundancy analysis (db-RDA) in vegan 2.3 (Oksanen et al., 2015), specifying a principal coordinates ordination of Bray–Curtis dissimilarities. Statistical significance of the terms in the db-RDA model was determined by 999 permutations of the distance matrix in vegan, restricting the permutations to within each individual to retain the nested structure of the data. The same procedure was also used to examine variation among tissue types in the abundance of KEGG pathways, except non-metric multidimensional scaling was used for ordination.

We assessed the difference in taxon abundance among tissue types in univariate analyses by fitting the data to a negative binomial generalized linear mixed model (GLMM) (Bates et al., 2013), specifying the individual ID as the random effect and the tissue type and animal source (field vs. lab) as fixed effects. A similar procedure was used to assess differences in 16S rRNA gene copy number, except a normal distribution was specified and technical replicates were nested within individual ID for the random effect. Likelihood ratio tests were used to determine the statistical significance of each factor (Venables and Ripley, 2002). Goodness-of-fit was assessed by with a chi-square test (Faraway, 2006) and homoscedasticity was assessed by examination of residual plots. Non-parametric methods were used in univariate analyses of the metagenomic predictions because no distribution provided a reasonable fit to the data. *P*-values were adjusted for multiple tests using the false discovery rate (Benjamini and Hochberg, 1995).

## Results

### Spatial Structure of the Gut Microbiome

**FIGURE 2:**
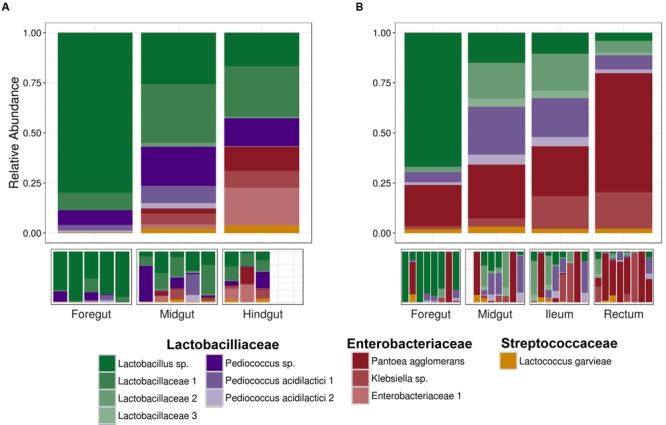
**Mean relative abundance of the eleven dominant sequence variants from (A)** field-caught and **(B)** laboratory-raised Mormon crickets from 16S rRNA Illumina sequencing. Note that field-caught and laboratory-raised animals differ in the DNA extraction method that was used.

We recovered 11 dominant sequence variants from field and lab-raised individuals (**Figure 2**), with the remaining 749 sequence variants comprising <1% of the sequences from a given Mormon cricket. Unassigned sequence variants (0.3%) and those assigned to mitochondria (2.9%) and chloroplast (1.6%) comprised a small proportion of the reads filtered prior to analysis. Field and laboratory-raised individuals shared 7 of the 11 sequence variants, including the most abundant *Pediococcus acidilactici* OTU that varied with mating status in a previous study (*P. acidilactici* 102222; Smith et al., 2016). The remaining five shared sequence variants were two Lactobacillaceae (*Lactobacillus* sp. and *P. acidilactici* 2), two Enterobacteriaceae (*Pantoea agglomerans* and a *Klebsiella* sp.), and one Streptococcaceae (*Lactococcus garvieae*). Field-caught Mormon crickets had three taxa that were not shared with laboratory-raised individuals (*Pediococcus sp*., *Lactobacillaceae 1, Enterobacteraceae 1*), while lab-raised individuals had one taxon (*Lactobacillaceae 3*) that was not shared with field individuals (**Figure 2**). Guts from two laboratory individuals were almost completely comprised of the enteric bacterium *P. agglomerans* (99.3% and 80.8% of reads, respectively), so we conducted our analysis with and without these individuals (hereafter referred to as “full” and “reduced” datasets).

**FIGURE 3:**
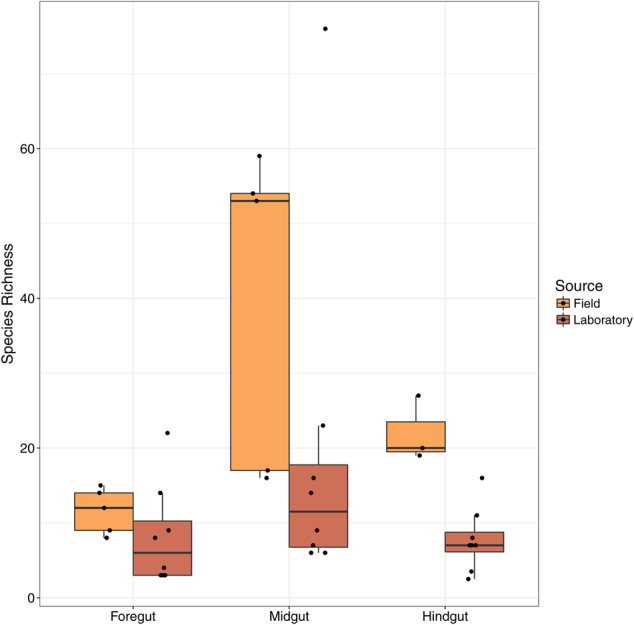
**Species richness among gut regions in Mormon crickets.** Note that field-caught and laboratory-raised animals differ in the DNA extraction method that was used.

Species richness (**Figure 3**) and diversity (Supplementary Figure S1) differed among gut regions and between field compared to lab-raised animals (**Table 1** and **Figure 3**). There was no significant interaction between collection source and tissue type (**Table 1**), indicating that differences in alpha diversity among tissue types were shared between lab and field-caught animals despite their differences in origin and DNA extraction protocol (see Materials and Methods). We found that the midgut was the most diverse with two of the three measures of alpha diversity (species richness and the Chao1 diversity estimator), while the hindgut and foregut had similar levels of richness and diversity. The foregut was also the least diverse region using the Shannon–Wiener index, but differed from the other two metrics in that the midgut and hindgut had similar levels of species diversity (Supplementary Figure S1).

**Table 1:** Analysis of deviance comparing alpha diversity between source populations (field or laboratory) and among tissue types (foregut, midgut, and hindgut).

	Reduced dataset			Full dataset		
	Species richness	Chao1	Shannon–Wiener	Species richness	Chao1	Shannon–Wiener
Source	**13.9 (0.003)**	**11.0 (0.007)**	**9.17 (0.01)**	**14.0 (0.002)**	**10.5 (0.006)**	**8.22 (0.013)**
Tissue type	**5.85 (0.010)**	**4.79 (0.02)**	**7.07 (0.005)**	**6.77 (0.004)**	**5.68 (0.008)**	**8.44 (0.001)**
Interaction	0.51 (0.61)	1.02 (0.38)	0.98 (0.39)	0.66 (0.53)	1.15 (0.33)	1.28 (0.29)

*Values represent the *F*-statistic (*p*-value) for each term. Statistically significant terms (*p* < 0.05) are indicated in bold. Degrees of freedom were estimated using the Kenward–Roger approximation. In the reduced dataset, two individuals from the laboratory-reared population were removed (see Materials and Methods).*

**FIGURE 4:**
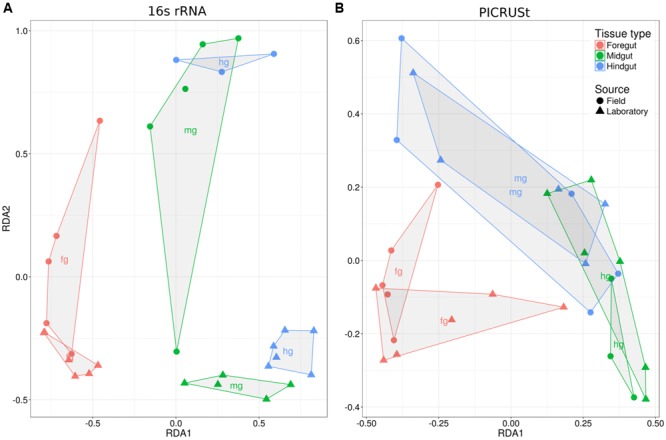
**Ordination of sample scores from the db-RDA of the reduced dataset for (A)** Illumina 16S rRNA sequencing and **(B)** PICRUSt metagenomic predictions. Note that field-caught and laboratory-raised animals differ in the DNA extraction method that was used.

The db-RDA analysis revealed that the structure of the gut microbiome also varied among gut regions (**Table 2** and **Figure 4A**). Like the species richness analysis, there was no significant interaction between tissue type and where the animals were sourced, indicating that differences in community structure among tissue types were consistent between field and laboratory-raised individuals (**Table 2**). To determine which members of the gut microbiome varied among gut regions, we plotted the taxa scores from db-RDA analyses of field and laboratory Mormon crickets (Supplementary Figure S2). Three groups of bacteria appeared to separate along the gut axis: a *Lactobacillus* sp. lactic-acid bacterium associated with the foregut, *Pediococcus* lactic-acid bacteria associated with the midgut, and *P. agglomerans*, an enteric bacterium, was found in association with the hindgut. Inspection of the plots from laboratory animals, where the ileum and rectum of the hindgut were dissected separately, indicate that *P. agglomerans* is relatively more abundant in the rectum, while the composition of the ileum, which is separated from the rectum by the colon, closely resembled that of the midgut (Supplementary Figure S2).

**Table 2:** Permutation tests of distance-based redundancy analyses of DADA2 16S rRNA sequence variants and PICRUSt metagenomic predictions.

	Reduced dataset		Full dataset	
	*F*	*P*	*F*	*P*
DADA2				
Source	**8.99**	**<0.001**	**7.75**	**<0.001**
Tissue type	**9.85**	**<0.001**	**5.49**	**<0.001**
Interaction	0.26	0.47	0.52	0.84
PICRUSt				
Source	0.23	0.76	**4.31**	**0.04**
Tissue type	**1.61**	**0.003**	**8.09**	**0.002**
Interaction	0.16	0.35	0.92	0.43

*Source population (field or laboratory) and tissue type (foregut, midgut, and hindgut) were entered as factors. Statistically significant terms (*p* < 0.05) are indicated in bold.*

Univariate analyses of these three groups largely confirmed the pattern in the ordination (**Table 3** and Supplementary Figure S3). The interaction between tissue type and source was not significant in any of the analyses and dropped to estimate the differences in relative abundance between tissue types. *Lactobacillus* sp. was three times more common in the foregut than in the midgut (β = 1.4 ± 0.50, *p* = 0.02) and seven times more common in the foregut than in the hindgut (β = 2.0 ± 0.51, *p* < 0.001). *Pediococcus* were similar in relative abundance in the midgut and hindgut (β = 0.65 ± 0.57, *p* = 0.49) but 4.7 times more common in these areas than the foregut (β = 1.1 ± 0.36, *p* = 0.006). *P. agglomerans* was 209 times greater in relative abundance in the hindgut than in the foregut (β = 3.8 ± 0.87, *p* < 0.001) and 12 times greater in relative abundance in the hindgut than in the midgut (β = 2.5 ± 0.82, *p* = 0.007).

**Table 3:** Likelihood ratio tests from GLMMs fitting the abundance of sequence variants to source population (field or laboratory) and tissue type (foregut, midgut, or hindgut).

	Reduced dataset			Full dataset		
	LAC1	PED	PAG	LAC1	PED	PAG
Source	1.32 (0.25)	0.80 (0.37)	2.36 (0.12)	0.18 (0.66)	0.51 (0.48)	0.01 (0.96)
Tissue type	**16.3 (<0.001)**	**9.25 (0.01)**	**20.7 (<0.001)**	**12.7 (0.002)**	**16.1 (<0.001)**	**41.9 (<0.001)**
Interaction	0.36 (0.84)	0.84 (0.66)	3.86 (0.15)	0.3 (0.86)	0.95 (0.62)	2.67 (0.26)

*Values are chi-square (*p*-value). LAC1, *Lactobacillus* sp.; PED, *Pediococcus*; PAG, *Pantoea agglomerans*. Statistically significant terms (*p* < 0.05) are indicated in bold.*

### Absolute Abundance of Bacteria from 16S rRNA qPCR

**FIGURE 5:**
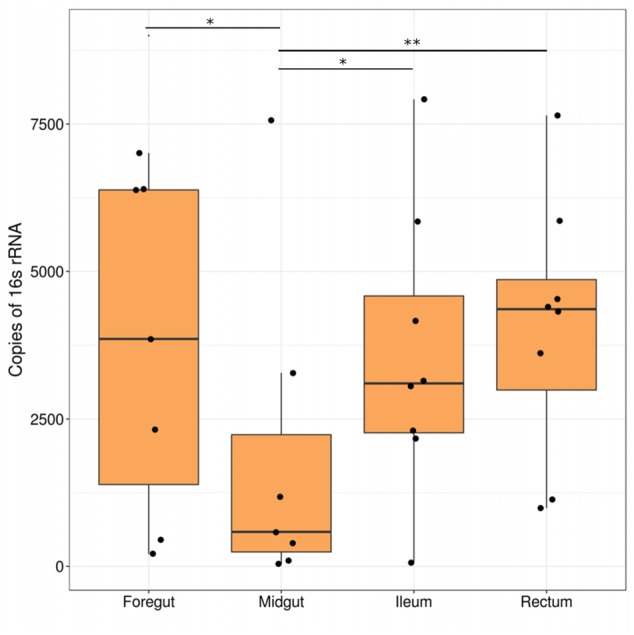
**Abundance of bacterial 16S rRNA genes in field-caught Mormon crickets.** Bars indicate significant differences between regions (^∗^*p* < 0.05, ^∗∗^*p* < 0.01).

The number of copies of bacterial 16S rRNA genes was significantly different among tissue types (analysis of deviance: *F*_3,64_ = 5.05, *p* = 0.003). The midgut had the lowest abundance of genomic 16S rRNA, while the foregut, ileum, and hindgut were not statistically distinguishable from each other (**Figure 5** and Supplementary Table S1). The average number of copies of 16S rRNA genes in the midgut was 50.6% lower than in the foregut, 47.6% lower than in the ileum, and 53.7% lower than in the rectum.

### PICRUSt Metagenomic Predictions

**FIGURE 6:**
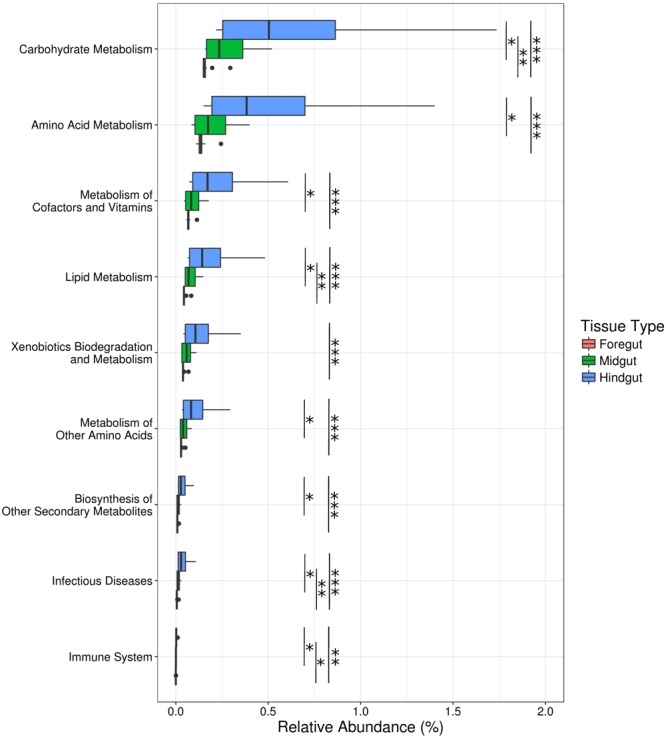
**Relative abundance of KEGG pathways related to nutrition, immunity, xenobiotic degradation, and secondary metabolite production among tissue types.** Sample sizes are foregut (*n* = 11), midgut (*n* = 11), and hindgut (*n* = 9). ^∗^*p* < 0.05, ^∗∗^*p* < 0.01, ^∗∗∗^*p* < 0.001.

PICRUSt analysis of 16S rRNA sequence variants recovered 5891 KEGG orthologs associated with 328 metabolic pathways. The representation of the predicted KEGG pathways differed significantly among gut regions in both the full and reduced datasets, while the source of the animals had a significant influence in the full dataset but not the reduced dataset (**Table 2** and **Figure 4B**). Neither analysis, however, showed an interaction between tissue type and whether an animal was wild or lab-reared, indicating that metagenomic predictions differed among tissue types in similar ways (**Table 2**). Univariate analyses found significant differences among tissue types in most KEGG pathways (Supplementary Table S2), including those that could affect host–microbe interactions via their role in nutrition, immunity, degradation of xenobiotics, and production of secondary metabolites (**Figure 6**). Among these functional groups, the hindgut exhibited the most abundant representation of each KEGG category, followed by the midgut and then the foregut (**Figure 6**).

### Nutrition

We searched our metagenomic predictions for specific bacterial genes known to contribute to host nutrition in orthopterans. We queried our database for enzymes capable of metabolizing the complex plant carbohydrates xylan, pectin, raffinose, and galactomannan, which are metabolized by gut bacteria in the house cricket *Achetus domesticus* (Kaufman and Klug, 1991), and cellulose, an important component of the plant cell wall. We found KEGG orthologs involved in the metabolism of all these complex plant polymers, except galactomannan. The pectin metabolic pathway was also incomplete. Only pectinesterase, the first enzyme involved in pectin metabolism, was found among the 11 dominant taxa in Mormon crickets, although enzymes involved in subsequent steps (e.g., polygalacturonase [EC:3.2.1.15], galacturan 1,4-alpha-galacturonidase [EC:3.2.1.67]) were represented in the minority members of the gut microbiome (i.e., <1% of 16S rRNA sequences, see Sequencing and Bioinformatics).

**FIGURE 7:**
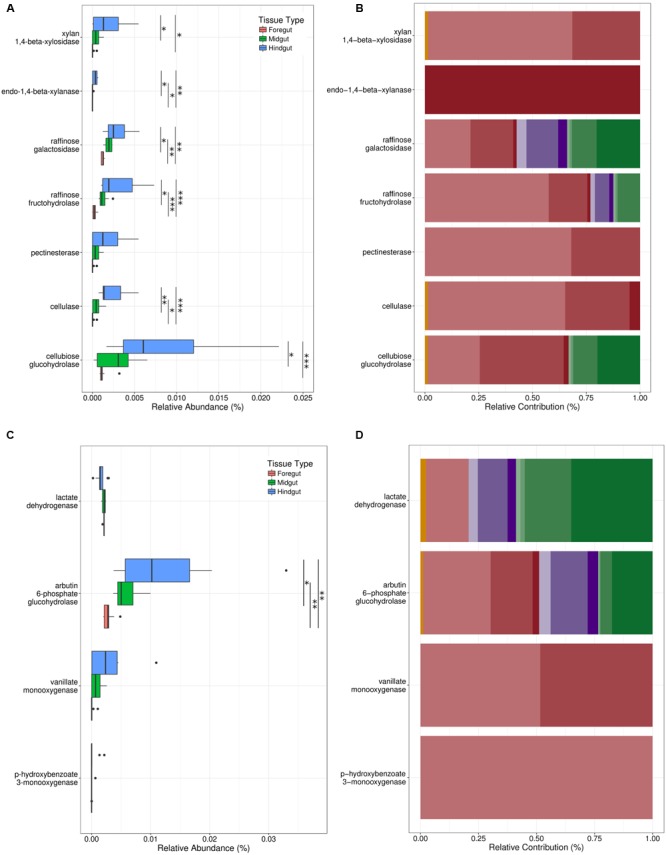
**Relative abundance of KEGG orthologs related to (A)** carbohydrate metabolism and **(B)** antimicrobial compound production among tissue types, and the contributions of taxonomic groups **(C,D)** to ortholog abundance. Key for colors representing taxonomic groups are in **Figure 2**. Sample sizes are foregut (*n* = 11), midgut (*n* = 11), and hindgut (*n* = 9). ^∗^*p* < 0.05, ^∗∗^*p* < 0.01, ^∗∗∗^*p* < 0.001.

The relative abundance of KEGG orthologs for carbohydrate metabolism in our samples were most pronounced in the hindgut (Supplementary Table S3 and **Figure 7A**) and dominated by the enteric bacteria, particularly *Klebsiella* sp. and Enterobacteriaceae 1 (**Figure 7B**). Lactic-acid bacteria, however, were also represented in predictions for raffinose metabolism and enzymes capable of participating in the hydrolysis of cellobiose to glucose via cellobiose glucohydrolase, but not cellulase, which hydrolyzes cellulose to cellobiose (**Figure 7B**).

Gut bacteria might also play a role in the production of the essential amino acid phenylalanine via the shikimate pathway, which is found in microbes and plants but not in animals (Herrmann and Weaver, 1999). Phenylalanine is required for stabilization of the cuticle following molting (Bernays and Woodhead, 1984) and is converted to tyrosine, the precursor of melanin, a key component of the insect immune response (González-Santoyo and Córdoba-Aguilar, 2012). All enzymes in the shikimate pathway were represented in our metagenomic predictions (Supplementary Figure S4), although prephenate hydrogenase, which is required for phenylalanine synthesis, was only represented in *Lactococcus garviae*. *L. garviae* abundance thus might influence the availability of phenylalanine for Mormon crickets, unless they are able to acquire it in sufficient quantities directly from their diet.

### Immunity

In the locust *Schistocerca gregaria* (Orthoptera), four phenols have been shown to increase resistance to microbial pathogens (Dillon and Charnley, 1988, 1995): hydroquinone, 3,4-dihydroxybenzoic acid, *p*-hydroxybenzoic acid, and 4,5-dihydroxybenzoic acid. We found enzymes in our metagenomic predictions associated with the production of all these compounds except for 4,5-dihydroxybenzoic acid, which was not annotated in the KEGG database. Hydroquinone production was represented by the enzyme arbutin 6-phosphate glucohydrolase, which metabolizes arbutin, a phenolic glycoside present in leaf and fruit tissue of many plants (Xu et al., 2015).

Two enzymes were found capable of producing 3,4-dihydroxybenzoic acid. The first, vanillate monooxygenase, demethylates vanillic acid, a compound derived from lignin (Bugg et al., 2011). This pathway was proposed in locusts based on the abundance of vanillic acid in their feces (Dillon and Charnley, 1988, 1995). The second enzyme, *p*-hydroxybenzoate 3-monooxygenase, oxidizes *p*-hydroxybenzoic acid, one of the other antimicrobial phenols in locusts (Dillon and Charnley, 1995). The most likely source of *p*-hydroxybenzoic acid in the diet of Mormon crickets is benzoic acid, which is a precursor to salicylic acid in plants (Raskin, 1992). The enzyme responsible for catalyzing the conversion of benzoic acid to *p*-hydroxybenzoic acid (benzoate 4-monooxygenase), however, was not found among the 11 dominant taxa in our samples, although it was present in the minority members of the Mormon cricket gut microbiome. Production of *p*-hydroxybenzoic acid in appreciable concentrations is thus less likely than for hydroquinone or 3,4-dihydroxybenzoic acid.

Like carbohydrate metabolism, the hindgut (**Figure 7C**) and enteric bacteria (**Figure 7D**) dominated the relative abundance of KEGG orthologs implicated in the production of antimicrobial phenols in our samples, with the exception of hydroquinone, which was represented to varying degrees among the lactic-acid bacteria. Notably, *P. agglomerans*, which has been reported to participate in the production of 3,4-dihydroxybenzoic acid in locusts (Dillon and Charnley, 1995), was not among taxa responsible for the occurrence of vanillate monooxygenase in our samples (**Figure 7D**).

Finally, we searched for three other known contributors to pathogen defense: bacteriocins and lactate dehydrogenase, which provides protection from pathogens in the gut by reducing pH (Servin, 2004). We found lactate dehydrogenase to be equally represented among gut regions (**Figure 7C**), and lactic-acid bacteria were the main contributors to our samples (**Figure 7D**). We found three bacteriocins in the KEGG database: nisin, mutacin, and *blp*-derived bacteriocins. None of these were found in our metagenomics predictions, perhaps not surprising considering their association with *Streptococcus*, which was not among the top 11 taxa in our samples (**Figure 2**). The bacteriocins we would expect to find based on taxonomy (e.g., pediocin for *Pediococcus*) were not annotated in the KEGG database.

We also found enzymes involved in the production of streptomycin, penicillin, and novobiocin in our metagenomic predictions, but not all enzymes required for their synthesis were present (data not shown). We did find β-lactamase, which confers resistance to β-lactam antibiotics (e.g., penicillins, cephalosporins, monobactams, and carbapenems; Drawz and Bonomo, 2010), represented among all the Enterobacteriaceae and *Pediococcus* taxa in our samples, but not among *Lactobacillus* sp. or other Lactobacillaceae (data not shown). This suggests that lactate production and antibiotic resistance could play a role in microbe–microbe interactions in the Mormon cricket gut microbiome.

### Phylogenetic Analysis of Cultured Isolates

Thirteen strains were cultured from the Mormon cricket gut based on 99% sequence similarity of their near full-length 16S rRNA genes (mean ± SD: 1406 ± 30 bp). Six were lactic-acid bacteria (Lactobacillaceae) and seven were enteric bacteria (Enterobacteriaceae).

**FIGURE 8:**
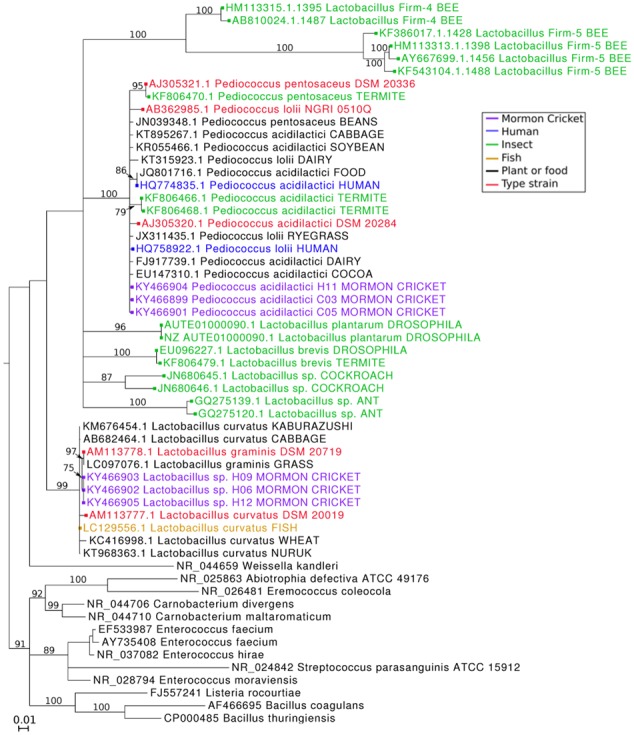
**Maximum likelihood estimation of phylogenetic relationships among lactic-acid bacteria 16S rRNA sequences from Mormon cricket gut isolates and their relatives.** Branches with bootstrap support <75% are collapsed.

The lactic-acid bacteria fell into two clades in our phylogenetic analysis (**Figure 8**). The first clade was comprised of *P. acidilactici* isolates derived from environmental sources, such as plants and various human foodstuffs, as well as strains from the human gut. Similarity to sequences from the BLAST search was high (>99.5%) and branch lengths were short, indicating that *Pediococcus* from the Mormon cricket gut are closely related to *Pediococcus* from environmental sources, unlike what has been found for *Lactobacillus* species isolated from bees (**Figure 8**; McFrederick et al., 2013).

Our search for *Pediococcus* sequences from insect guts in GenBank recovered sequences from the termites *Macrotermes bellicosus* and *Macrotermes subhyalinus*, which formed their own well-supported clade (**Figure 8**). Cultured *P. acidilactici* shared 100% sequence identity in the V4 region with the *P. acidilactici 1* phylotype sequenced using the Illumina platform in this study and with the *P. acidilactici* (102222) phylotype associated with variation in mating status in Mormon crickets (Smith et al., 2016). Morphologically, *P. acidilactici* were non-motile and spherical (0.8–1.0 μm), often dividing to form pairs as described for other *Pediococcus*. As other members of the genus, the *P. acidilactici* were Gram-positive, facultatively anaerobic, grow at low pH, and produce lactate from lactose (Supplementary Table S4).

The second clade of lactic-acid bacteria was comprised primarily of plant-associated *Lactobacillus*. Unlike *P. acidilactici*, these *Lactobacillus* formed a distinct clade with good branch support (**Figure 8**), indicating it is genetically distinct enough at the 16S rRNA locus to distinguish itself from other clades in the phylogeny. Similar to *P. acidilactici*, these *Lactobacillus* had high sequence similarity (>99.5%) to other members of the clade and a short branch length, indicating that while it is distinct enough to form its own clade, they are closely related to environmental sources of *Lactobacillus* at the 16S rRNA locus.

Our GenBank search for *Lactobacillus* isolated from insect guts found sequences from ants, bees, termites, and fruit flies, all of which fell into a different clade than *Lactobacillus* isolated from Mormon crickets. *Lactobacillus* from these taxa thus appear to have a different evolutionary history. *Lactobacillus* isolates shared 100% sequence identity in the V4 region with the Lactobacillaceae 2 phylotype sequenced using the Illumina platform in this study. Morphologically, these *Lactobacillus* appear as non-motile straight rods, approximately 1.3–2 μm in length and 0.8–1.0 μm wide and are Gram-positive, facultatively anaerobic, grow at low pH, and produce lactate from lactose (Supplementary Table S2).

**FIGURE 9:**
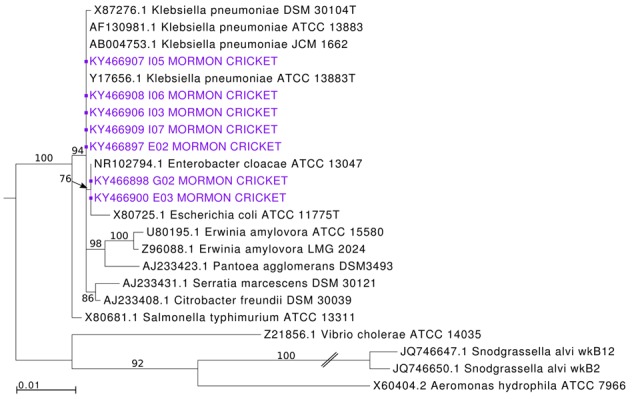
**Maximum likelihood estimation of phylogenetic relationships among enteric bacteria 16S rRNA sequences from Mormon cricket gut isolates and type strains.** Branches with bootstrap support <50% are collapsed.

The seven Enterobacteriaceae strains were most similar to *Enterobacter* strains in our BLAST search, which recovered sequences from a variety of plant and animal sources (sequence similarity = 98.7–99.8%). Our survey of GenBank found *Enterobacter* from alimentary tracts of a diverse group of insects, including termites, cockroaches, flies, beetles, stink bugs, bees, ants, and moths. Like other studies (Brenner et al., 2005), however, the 16S rRNA gene did not have enough signal to resolve relationships among *Enterobacter* and its relatives (data not shown) so we present a simpler phylogeny with the Mormon cricket isolates and type strains from the family (**Figure 9**).

We found that our Mormon cricket isolates were interspersed with *Enterobacter, Klebsiella*, and *Escherichia* type strains. A multilocus sequencing approach is thus needed to improve the inference (Brenner et al., 2005). All seven strains isolated from Mormon crickets had 100% identity at the V4 region with the *Klebsiella* phylotype sequenced on the Illumina platform, however, the phylogenetic (**Figure 9**) and phenotypic data (Supplementary Table S4) suggest that *Klebsiella* is unlikely to be a correct taxonomic assignment. Unlike most *Klebsiella*, cultured strains were motile, which is more typical of *Enterobacter* and other Enterobacteriaceae (Brenner et al., 2005). Morphologically, all isolates were straight rods, approximately 0.8–1.0 μm in length and 0.6–0.8 μm wide. Strains were Gram-negative and facultatively anaerobic (Supplementary Table S4).

## Discussion

We found striking differences in the diversity and structure of the gut microbiome in the Mormon cricket *A. simplex*. While most taxa were represented in the foregut, midgut, and hindgut, there were dramatic differences in relative abundance within the Lactobacillaceae and between the Lactobacillaceae and Enterobacteriaceae, the main families recovered in our culture and culture-independent studies. Predictions of their metabolic capabilities using PICRUSt suggest the potential for these gut bacteria to participate in the metabolism of complex carbohydrates and defense against microbial pathogens, particularly among the enteric bacteria in the midgut and hindgut, and to a lesser extent, the lactic-acid bacteria. Finally, our phylogenetic analysis of cultured isolates found that Mormon cricket gut bacteria are closely related to bacteria associated with plants or the guts of other animals, suggesting that gut bacteria are either acquired from the environment in each generation or have not been restricted to Mormon crickets over appreciable periods of evolutionary time.

Our finding that bacterial abundance is lower in the midgut is in agreement with reports from other orthopterans (Hunt and Charnley, 1981; Ulrich et al., 1981) and termites (Köhler et al., 2012), and has been attributed to characteristics that make the midgut less hospitable to bacteria than other regions of the alimentary tract (Douglas, 2015). The midgut in insects secretes a host of digestive enzymes, is immunologically active, and lined by the peritrophic membrane, which acts as a protective barrier that restricts microbes to the lumen and protects the epithelium (Douglas, 2015). In the two orthopterans that have been studied in detail, bacteria are found in the midgut lumen but not in association with the epithelium (Hunt and Charnley, 1981; Mead et al., 1988). As a consequence, midgut bacteria might need to be continually replenished from ingested food (Blum et al., 2013) because the peritrophic membrane is continually shed into the hindgut. In some insects, specialized midgut crypts provide niches that microbes colonize (Kikuchi et al., 2005; Bistolas et al., 2014). While the gastric caeca (**Figure 1**) have not been reported as an important site for microbial colonization in other orthopterans (Hunt and Charnley, 1981; Woodring and Lorenz, 2007), future studies should explore whether microbial communities in the caeca are similar in abundance and composition to the rest of alimentary tract.

The midgut is particularly vulnerable to pathogens because the lack of an endocuticle leaves the epithelium exposed once the peritrophic membrane is penetrated (Lehane and Billingsley, 1996). The Mormon cricket midgut was populated by lactic-acid bacteria, with *Pediococcus* specifically exhibiting greater relative abundance in the midgut (and hindgut) than in the foregut. Lactic-acid bacteria are known for their beneficial effects in insects, increasing resistance to parasites in bees (Forsgren et al., 2010) and promoting development in fruit flies by enhancing proteolytic activity (Erkosar et al., 2015) and upregulating host ecdysone and insulin-like peptides (Storelli et al., 2011). Lactic-acid bacteria are also known to suppress pathogenic bacteria by reducing pH through the production of lactate and by producing a number of antimicrobial compounds, such as hydrogen peroxide and bacteriocins (Cintas et al., 2001).

A previous study found that sexual interactions in Mormon crickets influences the relative abundance of three *Pediococcus* phylotypes (Smith et al., 2016), however, spatial information on where in the gut *Pediococcus* is located has been unavailable until now. *Pediococcus* in the midgut could provide immunological or nutritional benefits to Mormon crickets, as has been shown for *P. acidilactici* in other animals (Castex et al., 2008, 2009). We found that the capacity for lactate production from our metagenomics predictions was dominated by *Pediococcus* and other lactic-acid bacteria, although the relative abundance of the enzyme mediating lactate production was not higher in the midgut relative to other regions. The cultured isolates of *P. acidilactici* obtained from Mormon crickets in this study will enable future experimental and comparative genomic approaches to evaluate these hypotheses.

Lactic-acid bacteria were also common in the foregut, which was dominated by a *Lactobacillus* that averaged 73.9% of the sequences recovered from this region. Bignell (1984) noted that the foregut of insects tends to be the most acidic compartment, however, studies that measure the physiochemical environment and characterize microbiome composition of the foregut are rare (but see Köhler et al., 2012). This is because the endocuticle, lack of differentiated cells for absorption of nutrients, and frequent purging of consumed material into the midgut provides little opportunity for foregut microbes to contribute to host nutrition. The large differences in community structure between the foregut and the rest of the alimentary tract in our study does illustrate the dramatic transition in microbial communities between what is ingested and what can colonize the more distal regions of the gut. Our metagenomics predictions also suggest that the foregut is not the site of extensive carbohydrate metabolism or pathogen defense for most of the pathways we examined.

In contrast to the foregut and midgut, the hindgut was characterized by a dramatic increase in the relative abundance of enteric bacteria (Enterobacteriaceae). Ordination of the laboratory Mormon cricket samples indicated that the rectum, not the ileum, was primarily responsible for the difference in community structure in the hindgut (Supplementary Figure S2). Enterobacteriaceae comprised 83.5% of the sequences from the rectum compared to 57.5% from the ileum in laboratory-raised animals, which was more similar to the midgut in community structure (**Figure 2**). This distinction is potentially important because higher digestive efficiency in conventional compared to germ-free crickets has been attributed to microbial colonization of the ileum in the orthopteran *A. domesticus* (Kaufman and Klug, 1991).

Metabolism of the specific complex carbohydrates attributed to bacteria in Kaufman and Klug (1991) were also identified in our metagenomic predictions and localized to the midgut and hindgut, as well as enzymes involved in the production of the essential amino acid phenylalanine via the shikimate pathway. Phenylalanine is a precursor for tyrosine, which is required to stabilize the cuticle during molting (Bernays and Woodhead, 1984) and in phenoloxidase synthesis, an important component of the insect immune system (González-Santoyo and Córdoba-Aguilar, 2012). Tradeoffs between allocation of tyrosine to immune function and cuticle formation during development in Mormon crickets (Srygley, 2012) might be impacted by microbial contributions to amino acid production if sufficient quantities of these amino acids are not obtained directly from their diet.

Of the three enteric bacteria represented in this study, *P. agglomerans* was common to both field and lab individuals and increased in relative abundance in the hindgut. *Pantoea* are known plant pathogens and have been associated with a variety of medical conditions in humans (Walterson and Stavrinides, 2015). In insects, however, *Pantoea* have been shown to have mutualistic associations with their host. They are required for the completion of development in stinkbugs (Hosokawa et al., 2016; but see Dillon and Charnley, 2002), produce compounds that attract insects to their host plants in flies (Robacker et al., 2004; Maccollom et al., 2009), and in the orthopteran *S. gregaria*, produce a key component of the locust aggregation pheromone (Dillon et al., 2000, 2002) and reduce susceptibility to microbial pathogens through the production of phenols (Dillon and Charnley, 1986, 1995).

Our metagenomics predictions suggest that enteric bacteria in Mormon crickets might be capable of producing at least two of the antimicrobial phenols identified in *S. gregaria*, although *P. agglomerans* was not identified as an important contributor in our study. This illustrates a limitation of PICRUSt, as genomes in the IMG database used to make inferences about gene content may miss important among-strain variation in metabolic capabilities. *P. agglomerans* derived from *S. gregaria* are likely to have acquired this capability independently, unless the metabolic pathway is different from the one analyzed here or the taxonomic designation reported by Dillon and Charnley (1986, 1995) is outdated.

Metagenomic analyses are also dependent upon annotation of the relevant pathways in the KEGG database. We were unable, for example, to assess the potential for the Mormon crickets microbiome to produce bacteriocins or the aggregation pheromone guaiacol, a bacterial metabolite produced by *P. agglomerans* in *S. gregaria* (Dillon et al., 2000, 2002), because the predicted pathways are not annotated in the KEGG database. The role of the gut microbiome in protecting Mormon crickets from their own pathogens (MacVean and Capinera, 1991) and its influence on aggregation behavior (MacVean, 1987; Simpson et al., 2006) is thus an important direction for future research.

## Conclusion

Variation in morphology and physiology is thought to differentiate niches within the gut that influence the organization of the microbiome. Our study describes at high resolution how bacterial communities vary among gut regions, and suggests that host–microbe and/or microbe–microbe interactions have a role in how microbial communities are assembled and maintained. More detailed studies evaluating how microbial taxa are influenced by host physiology (Bignell, 1984; Ryu et al., 2010; Köhler et al., 2012), their distribution among microhabitats within gut regions (Kikuchi et al., 2005; Martinson et al., 2012), and experiments evaluating microbial community interactions (Cintas et al., 2001; Lee et al., 2013; Kwong and Moran, 2015) are needed to evaluate this possibility. While the metagenomics predictions gleaned from our study suggests that some of these bacteria might benefit Mormon cricket nutrition, immunity, and perhaps even modulate social behavior, experiments are needed to evaluate this possibility. Our establishment of methods for culturing Mormon cricket gut bacteria will enable experimental and comparative genomic approaches in the future to infer the ecological and evolutionary consequences of host–microbe symbiosis.

## Data Accessibility

Sequences and metadata are archived under NCBI BioProject PRJNA362233.

## Author Contributions

CS: Experimental design, Sample processing, data analysis, and MS preparation. RS: Field work and MS preparation. FH: Phenotyping and MS preparation. KS: Culture, sequencing and MS preparation. UM: experimental design, data analysis and MS preparation.

## Conflict of Interest Statement

The authors declare that the research was conducted in the absence of any commercial or financial relationships that could be construed as a potential conflict of interest.
