# Supplementary material for: Spatial Structure of the Mormon Cricket Gut Microbiome and its Predicted Contribution to Nutrition and Immune Function
Source: Front Microbiol. 2017 May 12;8:801. doi: 10.3389/fmicb.2017.00801 (PMC5427142; doi:10.3389/fmicb.2017.00801)
Supplement: Supplementary file 1 [file Data_Sheet_1.pdf]

SUPPLEMENTARY MATERIAL

DNA extraction and bioinformatics

Field-caught tissue samples were bead-beat in a sterile 2ml screw cap tube with 750 µl of 0.1mm silica-zircon beads (Biospec Products), two 3.2mm stainless steel beads (Biospec Products) and 1ml of sterile CTAB buffer (0.1M Tris, 1.4M NaCl, 20mM EDTA, 2% PVP, 2 µl beta-mercaptanol, 20µl of 20mg/ml Proteinase-K). Tubes were bead beat at maximum speed for 2 min (Mini Beadbeater-96, Biospec Products) and incubated overnight at 55°C. RNAse (400µg, Qiagen) was added to the lysate and incubated at 37°C for 30 min. A phenol-chloroform extraction with isopropanol precipitation was performed to isolate the DNA.

Library preparation was done by the Genome Sequencing and Analysis Facility at the University of Texas at Austin using the NEBNext kit and sequenced on an Illumina Miseq using V3 chemistry. Using DADA2, we trimmed the forward and reverse primers, truncated reads when Illumina quality scores were less than two, and removed sequences with a maximum expected error of one. Sample composition was then determined with DADA2, specifying joint inference of sample composition and sequence error rates (selfConsist=T). Chimeric sequences were removed on merged reads using DADA2’s removeBimeraDenovo algorithm and the remaining variants were classified using Greengenes 13.8 training set with DADA2’s assignTaxonomy function with the default parameters. Variants classified as unassigned, mitochondria, or chloroplast, and those that comprised an average of less than 1% of the reads recovered within a given Mormon cricket, were removed prior to analysis using phyloseq 1.16.2 (McMurdie & Holmes 2013).

Metagenomic prediction

The nearest sequenced taxon index (NSTI) was calculated in PICRUSt to determine the phylogenetic distance between our 16S rRNA sequences and the available genomes in the IMG database. NSTI values were small (mean + sd: 0.03 + 0.01, range: 0.006-0.040), indicating that the sequence variants identified by DADA2 from our 16S rRNA Illumina sequencing were closely related to the genomes available in the IMG database. PICRUSt metagenomic predictions of the human gut microbiome with similar NSTI values, for example, were positively correlated with empirical metagenomic analysis of those same samples (Spearman’s rho=0.72, Langille et al. 2013). We thus proceeded with the caveat that PICRUSt is a predictive tool to inform future experiments rather than a complete surrogate for true metagenomic sampling.

Table S1. Posthoc Tukey tests comparing the number of genomic 16s rRNA copies among gut regions in field-caught Mormon crickets (n=8). Values are the test statistic with the significance of the test indicated with an asterisk. FG=foregut; MG=midgut; ILE=ileum; REC=rectum.

	FG	MG	ILE	REC
FG	-			
MG	3.07*	-		
ILE	0.35	2.79*	-	
REC	0.43	3.57**	0.81	-

* p=<0.05, ** p<0.01

Table S2. Kruskal-Wallis tests for differences among gut regions in KEGG pathways. DF = 2 for all comparisons. P-values are corrected for multiple tests using the false discovery rate (FDR). Effect sizes (η^2) were calculated as (X^2-k+1)/(N-k) following Cohen (1998 p. 2).

**KEGG Pathway**	X^2	η^2	**p(FDR)**
Cell Growth and Death	19.7	0.654	0.0005
Metabolic Diseases	18.7	0.618	0.0005
Replication and Repair	18.6	0.616	0.0005
Translation	18.6	0.614	0.0005
Nucleotide Metabolism	18.6	0.614	0.0005
Nervous System	18.4	0.609	0.0005
Signaling Molecules and Interaction	18.3	0.604	0.0005
Lipid Metabolism	18.0	0.592	0.0005
Transcription	17.9	0.589	0.0005
Immune System Diseases	17.8	0.586	0.0005
Glycan Biosynthesis and Metabolism	17.7	0.582	0.0005
Poorly Characterized	17.5	0.575	0.0005
Infectious Diseases	17.5	0.573	0.0005
Carbohydrate Metabolism	17.2	0.564	0.0005
Cancers	16.9	0.552	0.0005
Genetic Information Processing	16.7	0.543	0.0006
Signal Transduction	16.2	0.527	0.0006
Membrane Transport	15.8	0.512	0.0008
Folding, Sorting and Degradation	15.6	0.504	0.0008
Excretory System	15.3	0.491	0.0009
Immune System	15.2	0.490	0.0009
Cell Motility	15.1	0.484	0.0009
Metabolism of Terpenoids and Polyketides	14.7	0.470	0.0010
Enzyme Families	14.5	0.462	0.0011
Environmental Adaptation	14.1	0.447	0.0013
Cellular Processes and Signaling	13.9	0.441	0.0014
Endocrine System	13.6	0.431	0.0015
Transport and Catabolism	12.7	0.395	0.0024
Biosynthesis of Other Secondary Metabolites	12.0	0.372	0.0031
Neurodegenerative Diseases	11.8	0.362	0.0034
Energy Metabolism	11.5	0.353	0.0036
Metabolism of Other Amino Acids	11.5	0.353	0.0036
Amino Acid Metabolism	11.4	0.347	0.0037
Metabolism of Cofactors and Vitamins	11.4	0.347	0.0037
Metabolism	10.7	0.322	0.0050
Xenobiotics Biodegradation and Metabolism	10.4	0.310	0.0057
Digestive System	2.6	0.024	0.2671

Table S3. Kruskal-Wallis tests for differences among gut regions in the relative abundance of KEGG orthologs related to nutrition and pathogen defense. DF = 2 for all comparisons. P-values are corrected for multiple tests using the false discovery rate (FDR). Effect sizes (**η²**) were calculated as (X^2-k+1)/(N-k) following Cohen (1998 p. 2).

KEGG ortholog	EC	X^2	η^2	p
Carbohydrate metabolism				
xylan 1,4-beta-xylosidase	3.2.1.37	8.51	0.24	0.024
endo-1,4-beta-xylanase	3.2.1.8	13.85	0.44	0.003
raffinose galactosidase	3.2.1.22	15.70	0.51	0.001
raffinose fructohydrolase	3.2.1.26	22.14	0.75	<0.001
pectinesterase	3.1.1.11	5.72	0.14	0.066
cellulase	3.2.1.4	18.81	0.62	<0.001
cellubiose glucohydrolase	3.2.1.21	11.86	0.37	0.005
Shikimate pathway				
3-deoxy-7-phosphoheptulonate synthase	2.5.1.54	12.30	0.38	0.005
3-dehydroquinate synthase	4.2.3.4	6.04	0.15	0.061
3-dehydroquinate dehydratase	4.2.1.10	2.83	0.01	0.255
shikimate dehydrogenase	1.1.1.25<br>1.1.5.8	15.85	0.24	0.001
shikimate kinase	2.7.1.71	9.88	0.29	0.013
3-phosphoshikimate 1-carboxyvinyltransferase	2.5.1.19	6.74	0.18	0.050
chorismate synthase	4.2.3.5	6.74	0.18	0.050
chorismate mutase	5.4.99.5	19.51	0.20	0.000
prephenate dehydratase	4.2.1.51	6.01	0.15	0.061
prephenate dehydrogenase	1.3.1.12	13.35	0.13	0.003
2-oxoglutarate aminotransferase	2.6.1.1<br>2.6.1.9<br>2.6.1.57	30.00	0.32	<0.001
Antimicrobial pathways				
lactate dehydrogenase	1.1.1.27	4.06	0.076	0.145
arbutin 6-phosphate glucohydrolase	3.2.1.86	19.64	0.653	<0.001
vanillate monooxygenase	1.14.13.82	6.31	0.159	0.059
p-hydroxybenzoate 3-monooxygenase	1.14.13.2	2.03	0.001	0.362

Table S4. Phenotypic description of Mormon cricket gut isolates. ND=not determined.

	**Lactobacilliaceae**						**Enterobacteriaceae**								
	**C03**	**C05**	**H06**	**H09**	**H11**	**H12**	**I3**	**I5**	**I6**	**I7**	**E02**	**E03**	**E06**	**G02**	**I09**
Gram Stain	+	+	+	+	+	+	-	-	-	-	-	-	-	-	-
Motility	0	0	0	0	0	0	1	1	1	1	1	1	1	1	0
Fermentation	1	1	1	1	1	1	1	1	1	1	1	1	1	1	1
Aerobic	1	1	1	1	1	1	1	1	1	1	1	1	1	1	1
Nitrate reduction	0	0	0	0	0	0	1	1	1	1	1	1	1	1	1
Gelatin hydrolysis	0	0	0	0	0	0	0	0	0	0	0	0	0	0	0
Citrate utilization	ND	ND	ND	ND	ND	ND	1	1	1	1	0	0	0	0	0
Catalase	0	0	0	0	0	0	1	1	1	1	1	1	1	1	1
Oxidase	0	0	0	0	0	0	1	1	1	1	0	0	0	0	0
Growth at:															
15C	1	0	1	1	1	1	ND	ND	ND	ND	ND	ND	ND	ND	ND
30C	1	1	1	1	1	1	1	1	1	1	1	1	1	1	1
35C	1	1	1	1	1	1	1	1	1	1	1	1	1	1	1
40C	1	0	1	1	1	1	1	1	1	1	1	1	1	1	1
45C	1	0	1	1	1	1	0	0	0	0	0	0	0	0	0
50C	0	0	0	0	0	0	0	0	0	0	0	0	0	0	0
55C	0	0	0	0	0	0	0	0	0	0	ND	ND	ND	ND	ND
pH 4.3	1	0	1	1	1	1	ND	ND	ND	ND	1	1	1	1	1
pH 7	1	1	1	1	1	1	ND	ND	ND	ND	1	1	1	1	1
pH 8.5	1	0	1	0	1	1	ND	ND	ND	ND	ND	ND	ND	ND	ND
NaCl 4%	1	1	1	1	1	1	ND	ND	ND	ND	ND	ND	ND	ND	ND
NaCl 6.5%	1	0	0	0	1	0	ND	ND	ND	ND	ND	ND	ND	ND	ND
NaCl 18%	0	0	0	0	0	0	ND	ND	ND	ND	ND	ND	ND	ND	ND

	Lactobacilliaceae						Enterobacteriaceae								
	**C03**	**C05**	**H06**	**H09**	**H11**	**H12**	**I3**	**I5**	**I6**	**I7**	**E02**	**E03**	**E06**	**G02**	**I09**
Decarboxylation of:															
Arg	ND	ND	ND	ND	ND	ND	1	1	1	1	1	1	1	1	1
Lys	ND	ND	ND	ND	ND	ND	1	1	1	1	1	1	1	1	1
Orn	ND	ND	ND	ND	ND	ND	1	1	1	1	1	1	1	1	1
MR	ND	ND	ND	ND	ND	ND	0	0	0	0	0	0	0	0	0
VP	ND	ND	ND	ND	ND	ND	1	1	1	1	1	1	1	1	0
H2S	ND	ND	ND	ND	ND	ND	0	0	0	0	0	0	0	0	0
Indole	ND	ND	ND	ND	ND	ND	0	0	0	0	0	0	0	0	0
Urease	ND	ND	ND	ND	ND	ND	0	0	0	0	0	0	0	0	0
Acid production from:															
fructose	1	1	1	1	1	1	1	1	1	1	1	1	1	1	1
galactose	1	1	1	1	1	1	1	1	1	1	1	1	1	1	1
glucose	1	1	1	1	1	1	1	1	1	1	1	1	1	1	1
lactose	1	1	1	1	1	1	0	0	0	0	1	1	1	1	1
maltose	1	1	1	1	1	1	1	1	1	1	1	1	1	1	1
ribose	1	1	1	1	1	1	1	1	1	1	1	1	1	1	1
sucrose	1	1	1	1	1	1	1	1	0	0	1	1	1	1	1
xylose	1	1	1	1	1	1	1	1	1	1	1	1	1	1	1

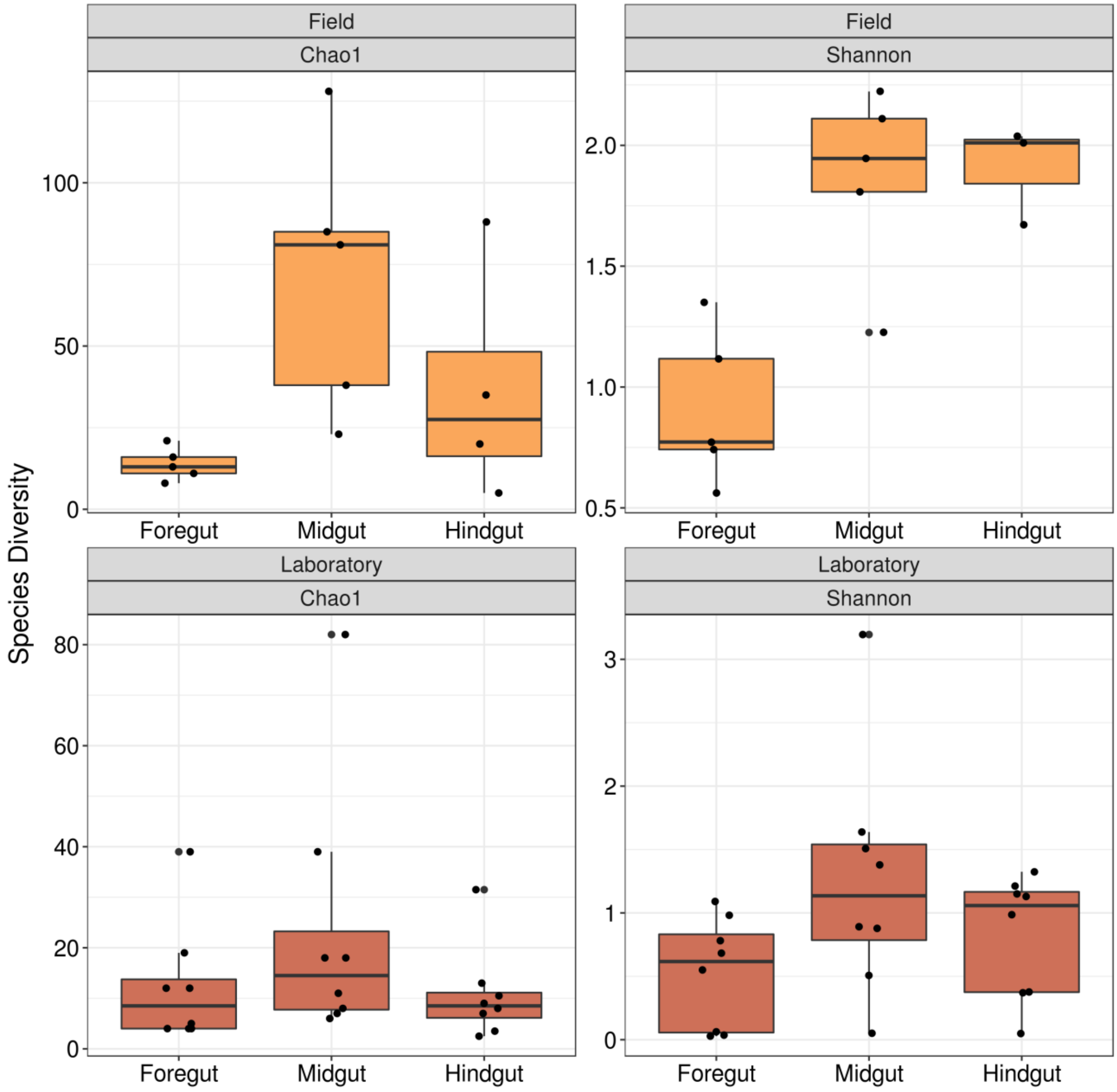

Fig S1. Species diversity among gut regions in Mormon crickets. Note that field-caught and laboratory-raised animals differ in the DNA extraction method that was used.

0

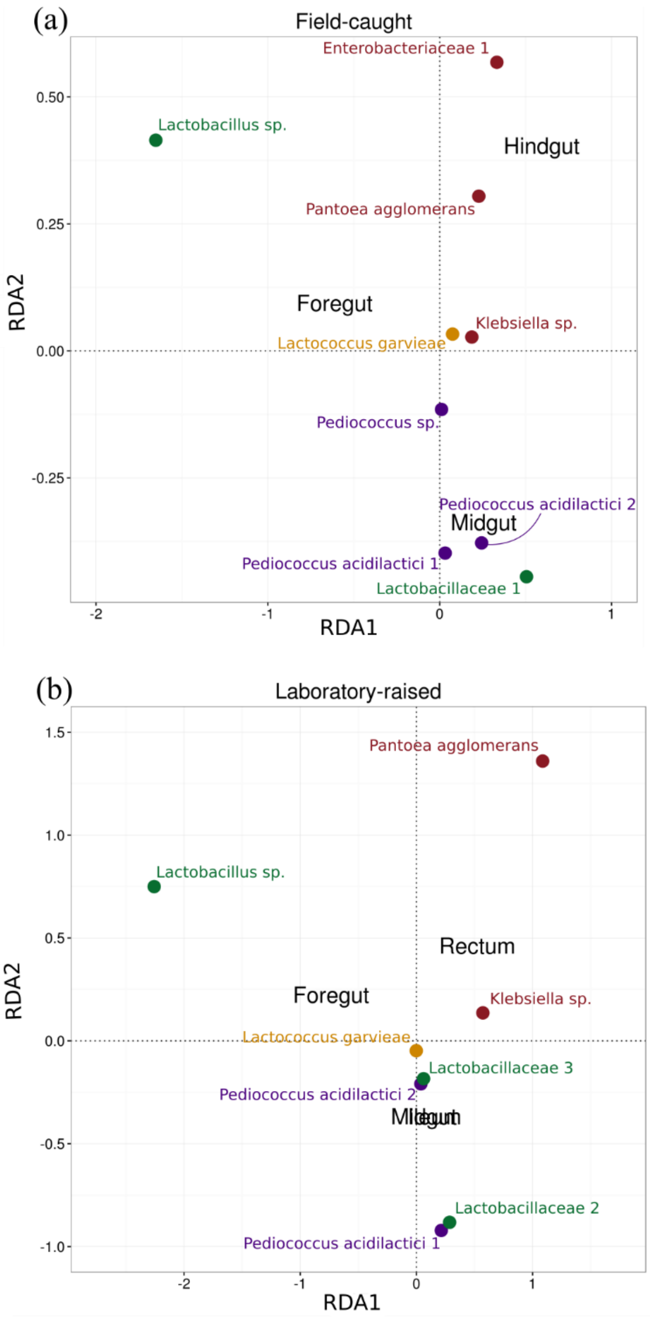

Figure S2. Ordination of sequence scores from the db-RDA of (a) field-caught and (b) laboratory-raised Mormon crickets. Means of sample scores for each tissue type are indicated in text [midgut and ileum centroids were similar and overlap in (b)]. Taxonomic groups are colored as in Figure 2.

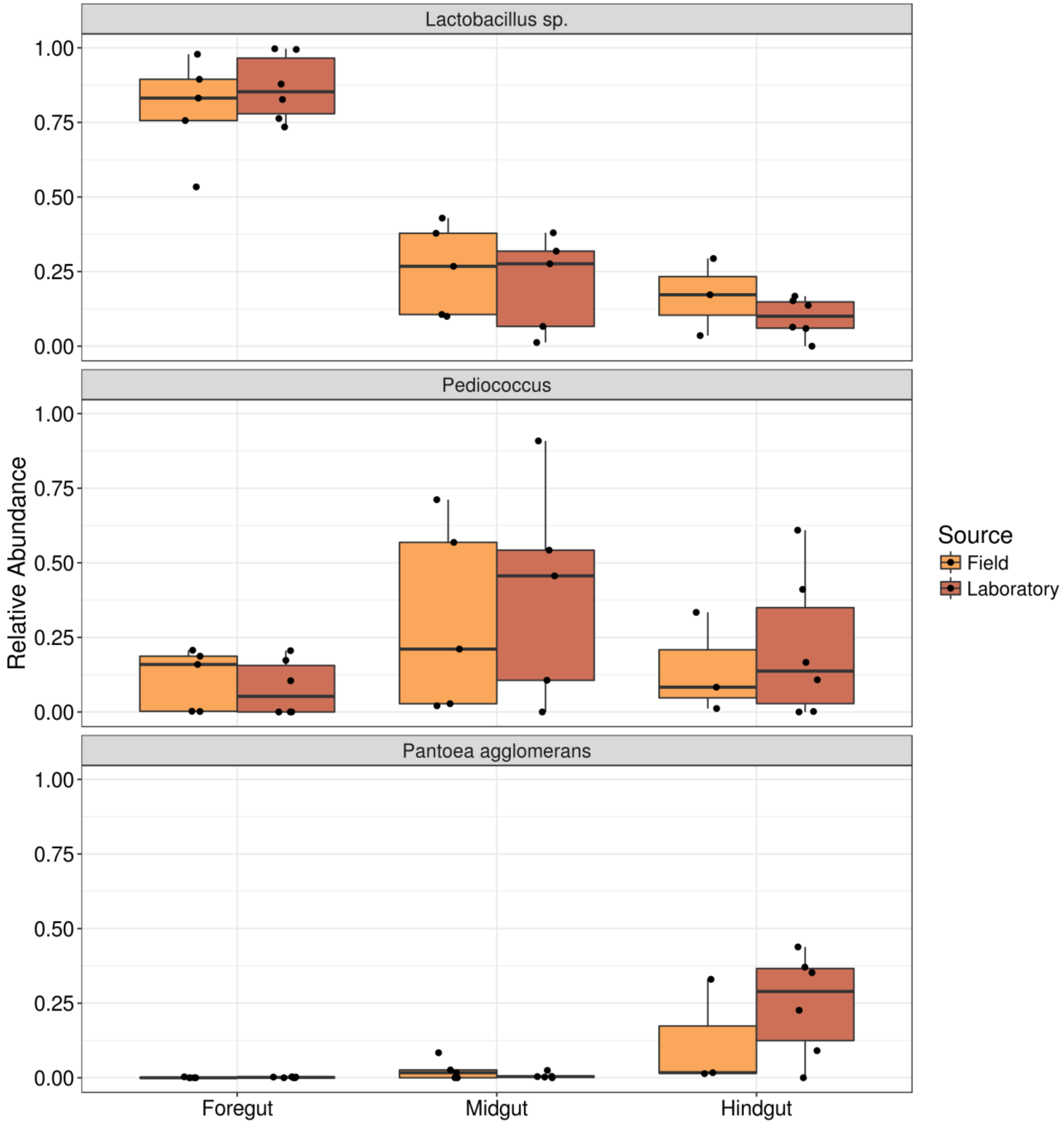

Figure S3. Relative abundance of *Lactobacillus sp.*, *Pediococcus*, and *Pantoea agglomerans* (reduced dataset) identified in the ordination (see Supplementary Figure 2) as associated with different gut regions. Note that field-caught and laboratory-raised animals differ in the DNA extraction method that was used.

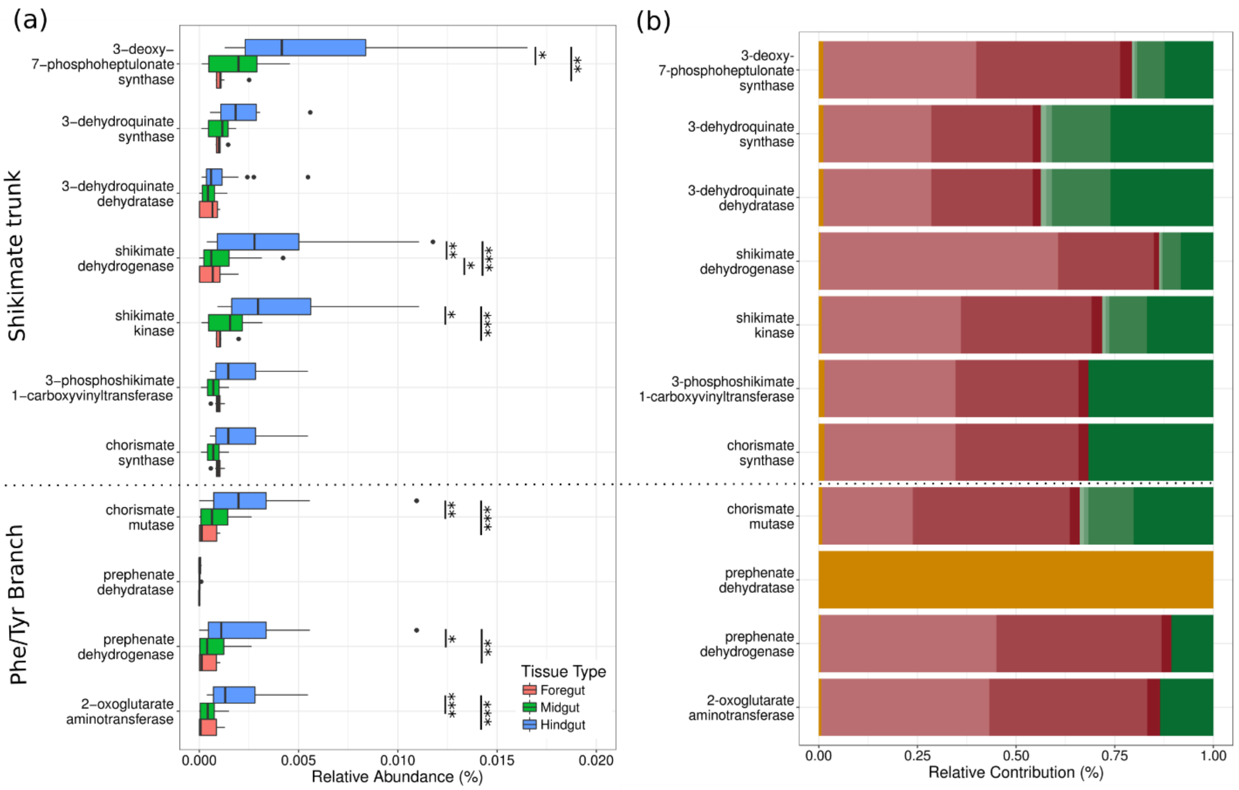

Figure S4. (a) Relative abundance of KEGG orthologs in the shikimate pathway (Fig. S5) and (b) the contributions of taxonomic groups to each ortholog as predicted by PICRUSt. Enzymes in the shikimate trunk are listed in the order that they occur in the pathway. Below the dotted line are the enzymes in the branch from the shikimate trunk to phenylalanine (phe) and tyrosine (tyr) synthesis. Key for colors representing taxonomic groups in (b) are in Figure 2. Sample sizes are foregut (n=11), midgut (n=11) and hindgut (n=9).

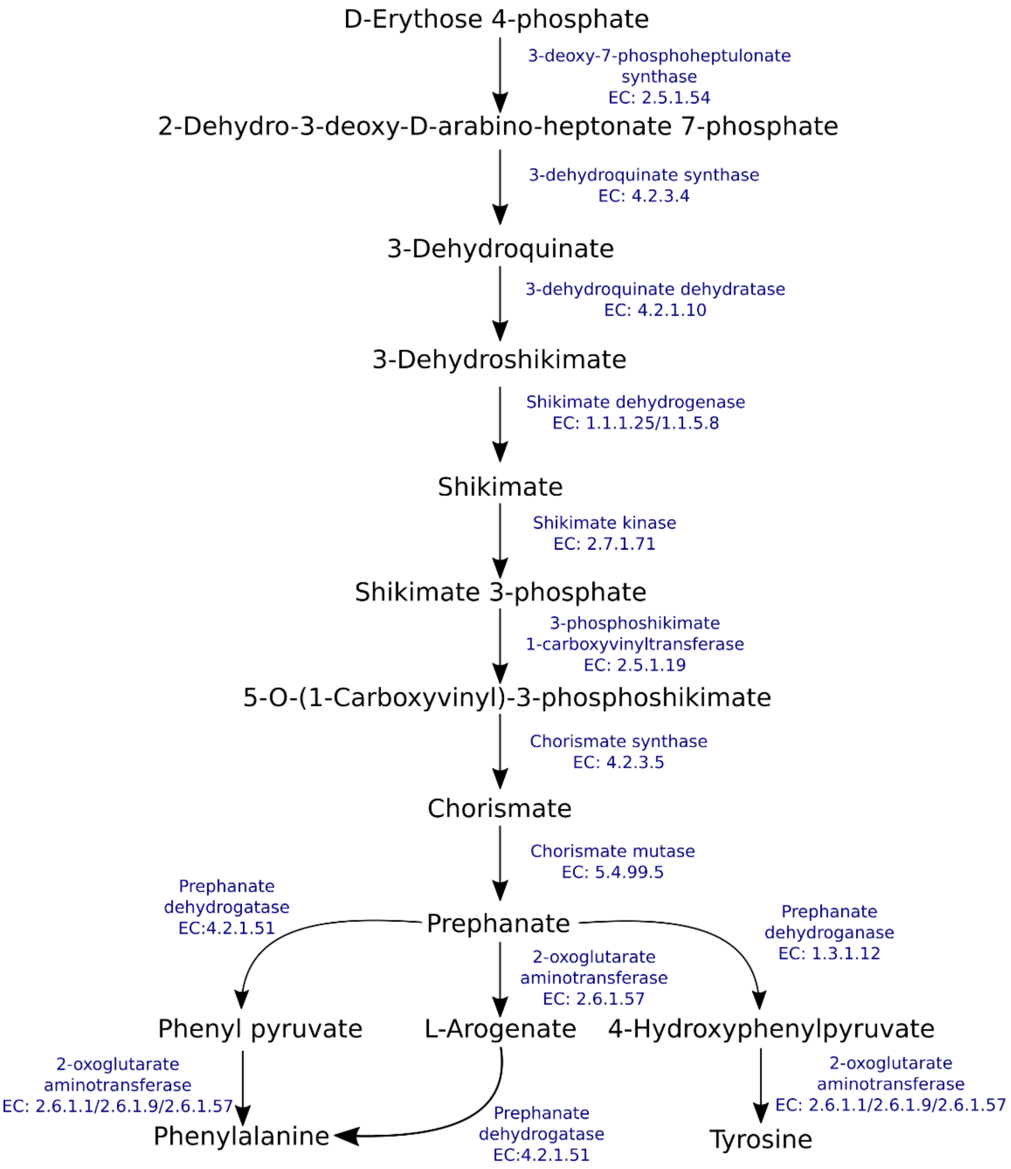

Fig S5. The Shikimate pathway. KEGG orthologs found in the PICRUSt metagenomic predictions are in blue. Sourced from http://www.genome.jp/kegg-bin/show_pathway?map00400+C00254.

References

Cohen, B.H. (1998) *Explaining Psychological Statistics*, 3rd ed. Wiley and Sons, New York.

Langille, M.G.I., Zaneveld, J., Caporaso, J.G., McDonald, D., Knights, D., Reyes, J.A., Clemente, J.C., Burkepile, D.E., Vega Thurber, R.L., Knight, R., Beiko, R.G. & Huttenhower, C. (2013) Predictive functional profiling of microbial communities using 16S rRNA marker gene sequences. *Nature Biotechnology*, **31**, 814–821.

McMurdie, P.J. & Holmes, S. (2013) Phyloseq: an R package for reproducible interactive analysis and graphics of microbiome census data. *PLoS ONE*, **8**, e61217.
